# Emerging roles of noncoding RNAs in idiopathic pulmonary fibrosis

**DOI:** 10.1038/s41420-024-02170-5

**Published:** 2024-10-21

**Authors:** Haitao Wang, Kai Sun, Hao Peng, Yi Wang, Lei Zhang

**Affiliations:** 1 Department of Pulmonary and Critical Care Medicine, NHC Key Laboratory of Respiratory Diseases, Tongji Hospital, Tongji Medical College, Huazhong University of Science and Technology, 1095 Jiefang Ave, Wuhan, 430030 China; 2 Xianning Medical College, Hubei University of Science & Technology, Xianning, 437000 Hubei China

**Keywords:** Non-coding RNAs, Respiratory tract diseases

## Abstract

Idiopathic pulmonary fibrosis (IPF) is a chronic, progressive, fibrotic lung disease with limited treatment options and efficacy. Evidence suggests that IPF arises from genetic, environmental, and aging-related factors. The pathogenic mechanisms of IPF primarily involve dysregulated repeated microinjuries to epithelial cells, abnormal fibroblast/myofibroblast activation, and extracellular matrix (ECM) deposition, but thus far, the exact etiology remains unclear. Noncoding RNAs (ncRNAs) play regulatory roles in various biological processes and have been implicated in the pathophysiology of multiple fibrotic diseases, including IPF. This review summarizes the roles of ncRNAs in the pathogenesis of IPF and their potential as diagnostic and therapeutic targets.

## Facts

- IPF is a fibrotic disease susceptible by epigenetic variations.
- Noncoding RNAs and other epigenetic mechanisms play a pivotal role in IPF.
- Interplay between noncoding RNAs and other epigenetic mechanisms in IPF pathogenesis remains largely unexplored.

## Open questions

- What are the specific molecular mechanisms by which dysregulated noncoding RNAs contribute to the development and progression of IPF?
- How can the potential roles of noncoding RNAs in mediating cross-talk between different cell types inform a better understanding of IPF?
- Can noncoding RNAs expression profiles serve as reliable biomarkers for IPF diagnosis, prognosis, and treatment monitoring?

## Introduction

Idiopathic pulmonary fibrosis (IPF) is the most common fibrosing lung disease and the most prominent type of idiopathic interstitial pneumonia and is characterized by chronic, progressive fibrosis [1]. The etiology of IPF is still unknown, and the disease is mostly irreversible and regarded as a significant public health burden due to its high mortality rate and lack of curative treatment options [2].

RNA, the transcription product of the genome of eukaryotes, including humans, can be divided into coding RNAs and noncoding RNAs (ncRNAs). The nucleotide sequence of the former can be translated into proteins to perform corresponding physiological functions. The latter, however, are not involved in coding proteins and can generally be divided into two categories: constitutive ncRNAs, such as tRNAs, rRNAs, and snRNAs, and regulatory ncRNAs, which are the focus of this review and include miRNAs, lncRNAs, circRNAs, etc.

ncRNAs are incapable of directly encoding proteins, but they possess the capacity to modulate cellular physiology and function through diverse mechanisms. These ncRNAs can influence normal gene expression patterns and have been found to play a vital role in both health and disease states and are novel therapeutic targets for pharmaceutical development and other interventions [3–6].

## Idiopathic pulmonary fibrosis

### Epidemiology

In recent years, the incidence and mortality rate of IPF have increased, and IPF is considered closely associated with aging [7–9], especially in high-incidence areas such as the United States, Canada, South Korea and Europe [7, 10, 11]. In the US and Europe, the incidence of IPF is estimated to be 3–17 per 100,000 person per year [12]. A study conducted among American veterans showed that the annual incidence increased from 73 to 210 cases per 100,000 person in 2010–2019 [13]. The prevalence and survival rates of IPF in Asia are generally lower than those in other parts of the world [14]. IPF is more common in men than women, and most of them are over 60 years old, with the peak of the disease occurring between 60 and 70 years of age [14]. Most patients with IPF have a median survival time of approximately 3 years after initial diagnosis [15], and the estimated survival time without treatment is 3–5 years, similar to that of cancer patients with a poor prognosis [1].

### Risk factors

It is now believed that IPF is the result of a combination of genetic and environmental factors. As a portal for gas exchange, the human respiratory system is exposed to microorganisms and various particles from the external and internal environment [16]. This exposure process is believed to be a key initiating factor in the progression of IPF by causing alveolar epithelial cell injury [17–20].

From a genetic perspective, the most common genetic variant in IPF is the MUC5B r35705950 allele [21], which may exacerbate injury or impair normal lung repair due to excessive mucin production and impaired mucociliary clearance. The second highest risk region identified is the desmoplakin gene, which is important for cell adhesion [22]. Additionally, epigenetic modifications mediated by miRNAs and lncRNAs may also contribute to the pathogenesis of fibrosis [23].

Aging is also a prominent risk factor for IPF, the prevalence of IPF doubles with every decade after age 50 [17]. At the cellular level, pulmonary fibroblasts and alveolar epithelial cells (AECs) in IPF have increased cellular senescence with higher expression of p21, p16 and p53 [24, 25]. Aberrant cellular senescence promotes fibrosis by impairing progenitor cell renewal and hindering repair of AECs and replacement of damaged lung tissue [26].

### The pathogenesis of IPF

**Fig. 1:**
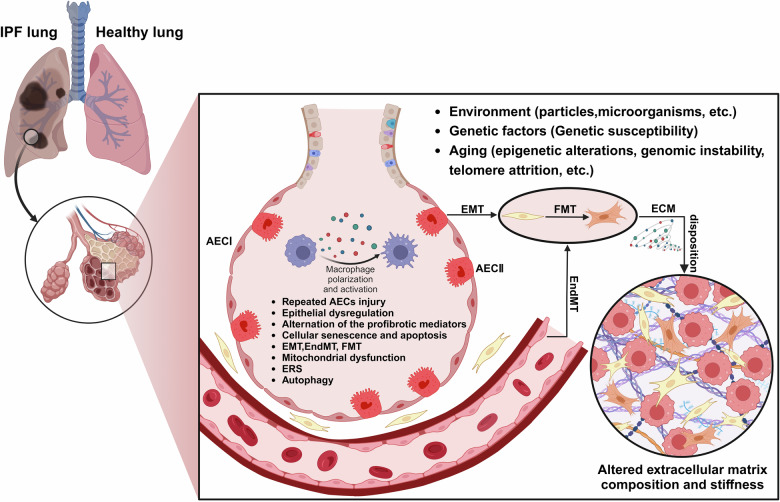
Overview of mechanisms contributing to the pathophysiology of IPF. Repeated injury to AECs leads to the dysregulation of their self-renewal and repair processes, accompanied by the activation of macrophages, which results in the aberrant secretion of cytokines and fibrogenic growth factors. This secretion recruits and activates fibroblasts and promotes their differentiation into myofibroblasts. These myofibroblasts produce excessive ECM and alter mechanical stiffness, triggering fibrosis and the remodeling of pulmonary architecture. (Created with BioRender.com).

The lung tissue constitutes a variety of cell types, including AECs, macrophages, fibroblasts, and myofibroblasts, which are involved in the development of IPF. It is generally accepted that the initiation and central process of IPF pathogenesis is the abnormal activation of type I alveolar epithelial cells (AECIs) following repetitive microinjuries [14, 27]. Specifically, when AECIs are subjected to microinjury, type II alveolar epithelial cells (AECIIs), which serve as lung-resident stem cells, promote the renewal of AECIs and facilitate the restoration of normal lung structure and function. However, after repeated lung injury, the repair capacity of AECIIs gradually decreases, leading to ineffective repair of the damaged alveolar epithelium [28]. Dysregulation of the wound healing process induces alveolar epithelial cells to secrete a cascade of mediators, including tumor necrosis factor (TNF)-α, interleukin (IL)-1, chemokine (CC-motif) ligand 2 (CCL2), connective tissue growth factor (CTGF), transforming growth factor β (TGF-β), and platelet-derived growth factor (PDGF), many of which are associated with fibrosis and subsequently promotes the proliferation of resident fibroblasts, fibroblast recruitment, and epithelial-mesenchymal transition (EMT), resulting in the formation of myofibroblast foci [29–31]. This further increases and modifies the extracellular matrix (ECM) and biomechanical stiffness [32]. The activation of myofibroblasts and the ECM in a positive feedback loop continuously drives the fibrotic process, culminating in an imbalance between profibrotic and antifibrotic effects [33, 34]. Currently, a growing body of evidence has proved the function of macrophages in pulmonary fibrosis (PF). Macrophages can produce numerous factors that regulate fibrosis and tissue repair. These macrophages undergo polarization into either M1 or M2 phenotypes. The interaction between these phenotypes has been considered to play a key role in the progression of IPF, and is of great significance for the severity and duration of the disease [35–37]. These alveolar macrophages secrete PDGF and other growth factors, promoting the activation and proliferation of fibroblasts, and even differentiation into myofibroblasts. In a positive feedback, fibroblasts secrete macrophage colony-stimulating factor (M-CSF) to maintain alveolar macrophages at the site of repeated injury [12, 38]. (Fig. 1).

In recent years, the signaling pathways and intracellular and extracellular processes involved in the IPF disease process, such as TGF-β1/Smad, Wnt/β-catenin, autophagy, endoplasmic reticulum stress, mitochondrial dysfunction, and EMT, have been confirmed to be increasingly associated with ncRNAs [17, 26, 39]. Next, we will discuss the various evidence supporting the potential role of ncRNAs in the pathogenesis of IPF, with respect to these key cells.

### Clinical manifestations

The most common symptoms of IPF are unexplained chronic exertional dyspnea and chronic dry cough [40]. Bibasilar inspiratory Velcro crackles are valuable for early diagnosis [41]. Digital clubbing occurs in 25–50% of cases, and signs of pulmonary hypertension and right ventricular failure may be seen in advanced stages [42–44]. Progressive deterioration of lung function characterizes IPF, which usually presents a restrictive pattern with forced vital capacity (FVC) and diffusion lung capacity for carbon monoxide (DL_CO_) [42].

### Diagnosis

IPF meets the histopathologic and/or radiologic pattern of usual interstitial pneumonia (UIP) [45]. Because of the strong correlation between radiologic and histologic manifestations of UPI, surgical lung biopsy (SLB) is recommended only for patients who have an HRCT pattern of probable UIP, indeterminate for UIP, or an alternative diagnosis. UIP pattern has been associated with other conditions, such as asbestosis and fibrotic hypersensitivity pneumonitis (fHP). Therefore, the exclusion of alternative diagnoses remains central [44]. The diagnostic criteria for IPF in the 2018 clinical guidelines published by TS/ERS/JRS/ALAT are as follows [45]: (1) Exclusion of other known causes of ILD (e.g., domestic and occupational environmental exposures, CTD, drug toxicity), and either (2) or (3); (2) The presence of the HRCT pattern of UIP; (3) Specific combinations of HRCT patterns and histopathology patterns in patients subjected to lung tissue sampling.

### Prognosis and staging

FVC is the most commonly used and easily measured prognostic indicator, and change in FVC over 6 to 12 months is highly predictive of outcome and superior to other baseline predictors [46]. DL_CO_, TLC, age, gender, smoking history, hypoxemia, comorbidities, radiologic findings and six-minute walk distance have also been reported to be associated with prognosis [47, 48]. Several staging systems can be used for prognosis assessment and staging, including the du Bois risk assessment system, the Ley GAP index and staging system, the Torrisi TORVAN predictive model, and the Cheng BRP prognostic model. These models integrate factors such as age, sex, lung function, and comorbidities to estimate patient mortality risk or transplant-free survival, thereby providing valuable reference for clinical decision-making [49–52].

### Treatment

The approval of pirfenidone and nintedanib for the treatment of IPF inaugurated the antifibrotic era. Pirfenidone and nintedanib also slows disease progression and reduces mortality. In patients with different baseline lung functions, they had similar efficacy and are equally effective in terms of IPF survival [53–55]. The most common adverse reactions of pirfenidone and nintedanib reported were gastrointestinal events and pirfenidone also caused skin-related adverse events [56, 57]. Pharmacological intervention can only slow the decline in lung function; lung transplantation is currently the only treatment that has the potential to improve quality of life and survival [58]. New compounds like nerandomilast, admilparant, inhaled treprostinil, and bexotegrast are evaluated in phase 3 or phase 2b trial stages [59]. Though current IPF medications are oral, inhalation routes offer targeted delivery, enhancing safety and efficacy but posing challenges in dose accuracy and potential respiratory tract changes [60, 61]. Furthermore, stem cell therapy shows promise but remains in exploratory stages, with current studies not yet demonstrating significant changes in lung function [62, 63].

## Noncoding RNA and IPF

### miRNAs

miRNAs are a group of short, single-stranded ncRNAs with a length of approximately 19 to 25 nucleotides. These miRNAs mediate RNA interference by binding to the 3′-UTR of target mRNA [64], which leads to either the cleavage of the complementary mRNA or the inhibition of its translation. miRNAs are involved in the regulation of apoptosis, proliferation and differentiation [65, 66]. In recent years, numerous reports have revealed the role of miRNAs in different stages of the development of IPF. In a 2010 study, researchers first identified alterations in the miRNA profile of IPF patients, many of which were subsequently shown to have potential roles in the progression of IPF [67]. Table 1 shows a list of dysregulated miRNAs in IPF.

**Table 1:** The role of miRNAs in IPF.

Function	miRNA	Experimental model	Target of action	Status in IPF	Effect on IPF or pathway associated	Reference
−	Let-7d	Mice/A549,FLF, NHLF, HFF-1	HMGA2 ER	Down-regulated	EMT and fibrotic markers by activation of TGFβ1/Smad3	[67] [72] [73]
−	miR-200	BLM fibrosis mice model and LPS early pulmonary fibrosis mouse model	TGF-β1 ZEB1/2	Down-regulated	TGF-β1-induced EMT of AECs	[70] [71]
+	miR-143-5p miR-342-5p	IPF fibroblasts and AECII	FASN ACSL-4	Up-regulated	TGFβ1/Smad3 pathway, ATII cell injury and senescence.	[76]
−	let-7	BLM fibrosis mice model	Sp3	Down-regulated	declined ferroptosis and improvement by Sp3/HDAC2/Nrf2 signaling pathway	[77]
			LOX1	Down-regulated	remits pulmonary fibrosis through regulating ROS, mtDNA damage, and NLRP3 inflammasome activation.	[78]
−	miR-29c	BLM fibrosis mice model and AEC2s of patients with IPF	Foxo3a	Down-regulated	Epithelial Cell Renewal and Apoptosis	[79]
		BLM fibrosis mice model	Fas	Down-regulated	ECM and the sensitivity to apoptosis in lung fibroblasts	[98]
−	miR-29b	Lung tissues of patients with IPF and BLM fibrosis mice model	TRIOBP	Down-regulated	EMT and lung fibroblast activation via TRIOBP/TRIO	[80]
−	miR-15a	BLM fibrosis mice model	YAP1	Down-regulated	fibroblast proliferation, migration, and collagen via YAP1/Twist	[82]
−	miR-338-3p	Lung tissues of patients with IPF and BLM fibrosis mice model	PTEN	Down-regulated	myofibroblast differentiation and matrix protein production via TGF-β/PTEN	[83]
−	miR-340-5p	NHLF-TGFβind	FNTATF1	Down-regulated	TGFβ/P38/ATF1 pathway	[84]
+	miR-301a	HFLT, HFLF-TGFβind and LF of BLM fibrosis mice model	TSC1	Up-regulated	Activation of TSC1/mTOR pathway	[85]
+	miR-21	BLM fibrosis mice model and HELF-TGFβind	Smad 7	Up-regulated	EMT and TGFβ/Smad pathway activation	[86]
+	miR-182-5p	BLM fibrosis mice model and HELF-TGFβind	Smad 7	Up-regulated	pro-fibrotic markers by pSmad2, pSmad3 and TGFβ pathway activation	[87]
−	miR-133a	NHLF-TGFβind	COL1A1 CTGF α-SMA TGFβR1	Up-regulated	TGF-β1-induced expression of classic myofibroblast differentiation markers	[90]
+	miR-124	LR-MSCs-TGFβind	AXIN1	Up-regulated	TGF-β1 induced differentiation of LR-MSCs to myofibroblast via Wnt signaling pathway	[91]
+	miR-424	NHLF-TGFβind and HFLF	SLIT2	Up-regulated	myofibroblast differentiation by TGFβ1 pathway	[92]
−	miR-17-5p	BLM fibrosis mice model	Thbs2	Down-regulated	Downstream fibrosis-related proteins	[94]
−	miR-34a	BLM fibrosis mice model	−	Down-regulated	diminished senescent phenotype and enhanced resistance to apoptosis	[95]
			FLIP	Down-regulated	myofibroblast resistance to T-cell initiated cell death and accumulation	[97]
+	miR-146a	Macrophages	IL-1R/TLRs-NF-κB axis, JAK-STAT signaling pathway	Up-regulated	M1 polarization ↓and M2 polarization ↑ , leading to fibrosis	[99]
+	miR-33	Macrophages	AMPK PGC-1α SIRT3	Up-regulated	mitochondrial homeostasis and augmentation of autophagy	[101]

#### miRNAs in AECs

Recent studies have shown that many miRNAs, including let-7d, miR-21, and miR-200, participate in the development of IPF through the regulation of EMT [67–71]. Previous studies have shown that the expression of let-7d is abnormally downregulated in IPF. This miRNA can increase profibrotic effects, including increasing the expression of various mesenchymal markers, such as N-cadherin-2, vimentin, α-SMA, and HMGA2, in multiple epithelial cell lines, thereby inducing EMT and promoting PF [67, 72, 73]. miR-21 expression is primarily increased in lung epithelial cells (LECs) isolated from PF mouse models and in AECs cultured under EMT-inducing conditions. The inhibition of miR-21 expression can reduce the expression of mesenchymal markers in AECs and delay EMT [69]. Research by Yang et al. found that members of the miR-200 family suppress EMT and reverse the fibrotic functions of lung fibroblasts. Moreover, as a negative regulator of TGF-β1, they can attenuate the expression of mesenchymal markers mediated by TGF-β1. The authors also noted that the restoration of miR-200c-3p may represent a novel approach for the treatment of PF, with potential significance for the diagnosis and treatment of IPF [70]. Additionally, in a study on the overlapping miRNA patterns in COVID-19 and IPF, two members of the miR-200 family, miR-200c-3p (upregulated) and miR-141-3p (downregulated), exhibited similar dysregulation in both COVID-19 and IPF [74]. Moreover, miR-200 family members make a difference in age-related IPF. On the one hand, transfection with miR-200 family members can restore the transdifferentiation capacity of senescent AECIIs into AECIs. On the other hand, some miR-200 family members can reduce the expression of senescence markers in AECIIs [75].

The role of miRNA-containing exosomes in the progression or treatment of IPF has gradually become better understood in recent years. Hayek et al. detected elevated levels of miR-143-5p and miR-342-5p in the exosomes of naive IPF patients. These miRNAs can downregulate the expression of FASN and ACSL-4 in AECIIs, leading to the injury and senescence of AECIIs [76]. Exosomes derived from menstrual stem cells (MenSCs) can deliver let-7 to MLE-12 cells to regulate the Sp3/HDAC2/Nrf2 axis, which ultimately suppressing ferroptosis and the progression of PF [77]. In addition to mediating the Sp3/HDAC2/Nrf2 signaling pathway, MenSC-derived exosomal let-7 has also been shown to alleviate PF by regulating ROS, mtDNA damage, and NLRP3 inflammasome activation [78].

Furthermore, recent studies have indicated that miRNAs have a potential regulatory role in the apoptosis of AECIIs during PF. Researchers have shown that miR-29c can inhibit the apoptosis of AECIIs by targeting and regulating the transcription factor Foxo3a, thus limiting the extent of lung tissue fibrosis [79].

#### miRNAs in fibroblasts/myofibroblasts

Numerous studies have demonstrated that miRNAs are capable of promoting the activation and proliferation of lung fibroblasts, which are key cells driving the pathological remodeling and fibrosis observed in IPF. Recent research by Wang et al. elucidated that miR-29b could regulate trio rho guanine nucleotide exchange factor (TRIO) by targeting F-actin binding protein (TRIOBP), thereby blocking EMT and lung fibroblast activation in IPF [80]. Chioccioli et al. demonstrated the antifibrotic activity of MRG-229, a miR-29 mimic, both in vitro and in vivo [81]. These findings suggest that promoting the expression of miR-29b may be a novel strategy for treating IPF. The expression of miR-15a was found to be significantly downregulated in IPF patients. Researchers discovered that miR-15a knockdown led to the overexpression of components of the YAP1/Twist axis, which promotes the proliferation, migration, and collagen production of lung fibroblasts. Conversely, the therapeutic restoration of miR-15a helped to ameliorate fibrosis [82]. Similarly, the expression of miR-338-3p was also downregulated in IPF, and considered to have antifibrotic potential. Transfection of primary human lung fibroblasts with miR-338-3p can induce the expression of PTEN (a known antifibrotic mediator that can suppress proliferation) and prevent the TGF-β1-mediated downregulation of PTEN, ultimately inhibiting myofibroblast differentiation and matrix protein production [83]. Another study revealed that miR-340-5p may be a negative regulator of IPF fibroblasts; its overexpression can alleviate the proliferation and activation of fibroblasts in PF by targeting ATF1 and inhibiting the TGF-β1-stimulated MAPK/p38 pathway [84]. In contrast, miR-301a expression was upregulated in mouse fibrosis models and IPF patients, and activated by TGF-β1 and IL-6 in fibroblasts. miR-301a negatively regulates TSC1 and activates the mammalian target of rapamycin (mTOR) signaling pathway. This promotes fibroblast activation and proliferation, myofibroblast formation, and collagen deposition. Genetic ablation of miR-301a or the intravenous administration of a miR-301a inhibitor can limit the progression of fibrosis [85].

TGF-β1 is a major fibrogenic factor in IPF, inducing the differentiation of lung fibroblasts into myofibroblasts, which involves changes in the levels of many classic myofibroblast differentiation markers. In 2010, the first miRNA investigated in a BLM-induced PF mouse model was miR-21. The upregulation of miR-21 expression could suppress the expression of Smad7 and reduce the phosphorylation of Smad2, thereby enhancing downstream TGF-β1 signaling events. This activation promoted fibroblast migration, proliferation, and differentiation into more myofibroblasts [86]. Another miRNA with profibrotic activity through the inhibition of Smad7 is miR-182-5p, which has been shown to be more highly expressed in TGF-β-stimulated human embryonic lung fibroblasts (HELFs) and in the lung tissues of fibrosis models [87]. In a 2018 study, the data indicated that the activated TGF-β1 signaling pathway can induce the expression of miR-21 [88]. Thus, there is a positive feedback loop between miR-21 and TGF-β1/Smad pathway. Blocking related fibrotic pathways with anti-miR-21 is also considered a promising therapeutic approach. For example, Yan et al. found that after lung-targeted delivery of cationic liposomes containing anti-miR-21, the differentiation of myofibroblasts and the synthesis of ECM were both suppressed [89]. Wei et al. reported that miR-133a, which is induced by TGF-β1, can act as a negative feedback regulator to downregulate the expression of classic myofibroblast differentiation markers [90]. In contrast to miR-133a, miR-424 and miR-124 have been validated as positive feedback regulators. Researchers have shown that miR-424, by reducing the expression of the slit2 protein (a protein that inhibits the pro-fibrotic signaling pathway mediated by TGF-β1), positively regulates myofibroblast differentiation, whereas miR-124 promotes the TGF-β1-induced differentiation of lung-resident mesenchymal stem cells (LR-MSCs) into myofibroblasts. Moreover, silencing miR-424 and miR-124 can reverse the upregulation of myofibroblast differentiation markers induced by TGF-β1 [91, 92].

Furthermore, Elevated miR-143-5p and miR-342-5p in naive IPF patients’ exosomes induce profibrotic responses by upregulating Smad3 and TGF-β1 in fibroblasts [76]. Exosomes derived from embryonic stem cells (ESC-exos) have also received increasing amounts of attention [93]. In a BLM-induced IPF model, human embryonic stem cells (hESCs)-exo-derived miR-17-5p can downregulate the transcription of Thbs2 in the cell nucleus to reduce the expression of downstream fibrosis-related proteins and suppress inflammation and fibrosis [94].

Recent studies have indicated that miRNAs engage in the apoptosis of fibroblasts and myofibroblasts in IPF. A 2017 study revealed that lung fibroblasts from miR-34a-deficient mice exhibited a decreased senescent phenotype and enhanced resistance to apoptosis [95]. Subsequently, in IPF lung tissues and myofibroblasts, the levels of miR-34 were inversely correlated with the expression of the survival molecule FLICE-like inhibitory protein (FLIP), which mediates the shift of myofibroblasts from death and apoptosis toward proliferation [96, 97]. Studies have also shown that the introduction of miR-29c mimics increased the death receptor Fas protein levels and induced apoptosis, restoring the normal sensitivity of lung fibroblasts to apoptosis [98].

#### miRNAs in macrophages

The study of miRNAs within macrophages in lung fibrosis, including IPF, is ongoing. Depending on the microenvironment, macrophages can polarize into a classical activation state (M1) or an alternative activation state (M2). M1 macrophages are responsible for wound healing after alveolar epithelial injury, while M2 macrophages are associated with the wound healing process, including fibrosis, or the termination of the lung inflammatory response [37, 99]. Liao et al. reported that miR-146a suppresses the M1 polarization of macrophages and promotes M2 polarization. This may lead to increased fibrosis but also exerts anti-inflammatory effects [99]. This apparent contradiction may be due to the role of miR-146a in maintaining the balance between the two states [100]. Furthermore, studies have shown that miR-33 is upregulated in bronchoalveolar lavage (BAL) cells isolated from patients with IPF. And specific genetic ablation of miR-33 in macrophages has been demonstrated to improve mitochondrial homeostasis and increase autophagy, shifting macrophages from a profibrotic to an antifibrotic phenotype, leading to the regression of fibrosis and reduced inflammation and PF after BLM injury [101]. Additionally, miR-29a, miR-185, miR-142-5p, miR-130a-3p, and miR-155 have also been found to potentially have profibrotic effects on macrophages [102–104].

### lncRNAs

lncRNAs are a class of ncRNAs that contain more than 200 bp and do not encode proteins. The underlying mechanisms involve epigenetic modification, transcriptional and post-translational regulation. In addition, lncRNAs can act as sponges for miRNAs, blocking their effects, and can even serve as precursors of other small RNAs (such as miRNAs and piRNAs) [105, 106]. Recent studies have shown that several lncRNAs are dysregulated and participate in fibrosis [105, 107]. With the continuous development of research, the role of lncRNAs in IPF is receiving increasing attention. Researchers identified 26 underexpressed and 49 overexpressed lncRNAs in the IPF through a meta-analysis of RNA-seq data. KEGG pathway analysis revealed that these RNAs are involved in several biological processes, including inflammation, compound metabolic processes, and DNA double-strand break repair [108]. Therefore, the significance of monitoring lncRNA level in vivo and their potential as therapeutic targets cannot be ignored. Table 2 shows a list of dysregulated lncRNAs in IPF.

**Table 2:** The role of lncRNAs in IPF.

Function	lncRNA	Experimental model	Target of action	Status in IPF	Effect on IPF and pathway associated	Reference
+	lncRNA MIR100HG	BLM fibrosis mice model	miR-29a-3p Tab1	Up-regulated	miR-29a-3p↑and Tab1↓	[109]
+	lncRNA SNHG8	BLM-induced A549 cells	miR-4701-5p	Up-regulated	pulmonary fibrosis via TGF-β1/Smad2/3 pathway	[110]
+	lncRNA TUG1	BLM fibrosis mice model	TGF-β1	Up-regulated	attenuating inflammation, EMT, inducing autophagy and inactivating PI3K/Akt/mTOR pathway	[113]
+	lncRNA TERRA	BLM fibrosis mice model	telomeric and mitochondria	Up-regulated	Improvement of telomeric and mitochondrial functions	[114]
+	lncRNA-ATB	BLM fibrosis mice model and TGF-β1-treated A549 cells	miR-200c/ZEB1	Up-regulated	promoting EMT via miR-200c/ZEB1	[116]
+	lncRNA ZEB1-AS1	BLM fibrosis mice model and TGF-β1-induced RLE-6TN cells	miR-141-3p	Up-regulated	E-cadherin, α-SMA, and EMT	[117]
+	lncRNA DANCR	BLM fibrosis mice model and TGF-β1-induced RLE-6TN cells	AUF1/FOXO3	Up-regulated	EMT-related protein changes via AUF1/FOXO3	[118]
+	lncRNA DNM3OS	BLM fibrosis mice model	miR-199a-5p miR-214-3p miR-199a-3p	Up-regulated	three pro-fibrotic miRNAs↑ (miR-199a-5p, miR-199a-3p, and miR-214-3p)	[119]
		HFLF-TGFβind	EZH2/TSC2	Up-regulated	promoting fibrosis in human embryonic lung fibroblasts via EZH2/TSC2	[120]
+	lncRNA Hoxaas3	BLM fibrosis mice model and TGF-β1-induced fibroblasts	Runx1 miR-450b	Up-regulated	activation of fibroblast via Runx1/miR-450b	[121]
+	LINC00941/lncIAPF	Lung tissues of patients with IPF and BLM fibrosis mice model	ELAVL1/HuR	Up-regulated	FMT, the activation of myofibroblasts via inhibiting autophagy	[123]
−	lncRNA FENDRR	Asbestos-Induced fibrosis mice model	SRSF9	Down-regulated	Suppressing the translation of β-catenin and fibroblast proliferation via SRSF9/mTOR	[124]
−	lncRNA GAS5	BLM fibrosis mice model	KDM5B	Down-regulated	the inhibition of pericyte-myofibroblast transformation	[127]
+	lncRNA-LINC000665	BLM fibrosis mice model	miR-214-3p /XBP1	Up-regulated	ERS-mediated IPF via miR-214-3p /XBP1	[129]

#### lncRNAs in AECs

Past studies have shown that there is a strong link between TGF-β1 and lncRNAs. In both BLM-induced PF and TGF-β1-stimulated MLE-12 cells, the lncRNA MIR100HG was aberrantly upregulated. However, MIR100HG knockdown directly upregulated miR-29a-3p and subsequently downregulated Tab1, which attenuated the TGF-β1-induced fibrotic changes [109]. Zhang et al. reported that the lncRNA SNHG8 can target miR-4701-5p to upregulate MUC5B expression, thereby promoting the progression of IPF [110]. They also found that SNHG8 overexpression enhanced the levels of TGF-β1 and phosphorylated Smad2/3 (p-Smad2/3). Considering that TGF-β1 levels are significantly decreased in MUC5B-deficient mice [111] and that a miR-4701-5p inhibitor also helps to increase TGF-β1 and p-Smad2/3 levels, the SNHG8/miR-4701-5p/MUC5B axis appears to regulate PF by modulating the TGF-β1/Smad2/3 signaling pathway.

Decreased autophagy in epithelial cells is a feature of IPF. Multiple studies have shown that PI3K/Akt/mTOR-dependent autophagy plays an important role in IPF [110, 112]. The knockdown of the lncRNA TUG1 can suppress the activation of the PI3K/Akt/mTOR pathway induced by TGF-β1 in RLE-6TN cells and can also ameliorate BLM-induced PF in rats [113].

Telomeric repeat-containing RNA (TERRA) is a type of lncRNA. Gao et al. reported that TERRA expression was nearly 4-fold greater in IPF patients than in controls and was negatively correlated with FVC as a percentage of the predicted value [114]. The knockdown of TERRA improved mitochondrial function and increased the expression of antioxidant enzymes such as catalase and superoxide dismutase, thereby exerting protective effects against oxidative stress.

ZEB1 is a key mediators of EMT and promotes lung fibrosis in an EMT-dependent manner [115]. Guan et al. reported that the upregulation of lncRNA-ATB expression inhibits the negative regulation of ZEB1 by competitively binding to miR-200c to promote EMT [116]. In RLE-6TN cells, the lncRNA ZEB1-AS1 acts as a competitive endogenous RNA (ceRNA) for miR-141-3p, increasing ZEB1 expression and facilitating the fibrotic process. ZEB1-AS1 knockdown reverses BLM-induced downregulation of E-cadherin expression and upregulation of α-SMA expression, indicating the suppression of EMT [117]. Furthermore, the lncRNA DANCR has been shown to induce EMT-related protein changes in RLE-6TN cells, which is achieved through the recruitment of AU-binding factor 1 to activate the translation of FOXO3 mRNA [118].

#### lncRNAs in fibroblasts/myofibroblasts

As a repository for “FibromiRs”, the lncRNA DNM3OS generates three different profibrotic miRNAs that regulate the TGF-β1 pathway [119]: (1) miR-199a-5p downregulates CAV1, impairing the degradation of the TGF-β/TGF-βR complex; (2) miR-214-3p promotes the noncanonical GSK-3β/β-catenin axis of TGF-β1 signaling by targeting COX-2 and GSK-3β; (3) the upregulation of miR-199a-3p suppresses the release of the antifibrotic factors FGF7 and HGF in response to TGF-β1. Another study revealed that DNM3OS can recruit EZH2 to the promoter region of the fibrosis suppressor TSC2, suppressing its expression and promoting fibrosis in HELFs [120].

Lin et al. revealed the mechanism by which the TGF-β1/Smad4-Hoxaas3-miR-450b-5p-Runx1 axis regulates IPF [121]. The expression of the lncRNA Hoxaas3, a transcriptional target of the TGF-β1/Smad4 pathway, is upregulated in the lungs of mice with PF. Aberrantly elevated Hoxaas3 levels increase Runx1 (runt-related transcription factor 1) levels and promote the activation and fibrosis of lung fibroblasts by negatively regulating miR-450b.

Defective autophagy has been shown to contribute to the activation and generation of myofibroblasts [122]. Zhang et al. demonstrated that the highly expressed lncRNA lncIAPF forms an RNA‒protein complex with ELAVL1/HuR (ELAV-like RNA binding protein 1) and regulates the stability of its target genes EZH2, STAT1 and FOXK1 to suppress autophagy [123]. Their research indicated that highly upregulated lncIAPF expression promotes the differentiation of fibroblasts into myofibroblasts, as well as the proliferation and migration of myofibroblasts, by inhibiting autophagy in the context of PF.

β-catenin is a pro-proliferative molecule, and serine-arginine rich splicing factor 9 (SRSF9) enhances its synthesis in an mTOR-dependent manner. Compared to that in normal lung fibroblasts, the expression of the lncRNA FENDRR is decreased in IPF fibroblasts [124]. By binding to SRSF9 and influencing downstream signaling pathways, including the mTOR pathway, the lncRNA FENDRR can reduce the translation of β-catenin and suppress fibroblast proliferation. Thus, low expression of the lncRNA FENDRR in fibrotic lung fibroblasts may be a contributing factor to enhanced cell proliferation.

PDGF helps fibroblasts migrate into injured lungs [125], and nintedanib exerts its antifibrotic effects by inhibiting PDGFRα/β [126]. Wang et al. reported that the lncRNA GAS5 acts as a scaffold to recruit KDM5B to the PDGFRα/β promoter, which leads to the inhibition of pericyte-myofibroblast transformation by suppressing PDGFRα/β expression through H3K4me2/3 demethylation [127].

X-box binding protein 1 (XBP1) is a downstream effector of inositol-requiring enzyme 1α (IRE1α) activation and is an integral component of the unfolded protein response (UPR) pathway [128]. Song et al. reported that elevated levels of the lncRNA LINC00665 in lung fibroblasts increase XBP-1 expression by targeting miR-214-3p, contributing to endoplasmic reticulum stress (ERS)-mediated IPF [129].

### circRNAs

circRNA is a long single-stranded ncRNA and have a covalently closed-loop structure [105, 130]. Emerging evidence suggests that circRNAs function as gene regulators in mammals, particularly by acting as miRNA or protein sponges, mRNA translation templates, protein scaffolds and function enhancers [131–134]. Disruption of the regulation of circRNAs can impact multiple cellular processes and signaling pathways [135], thus contributing to the occurrence and development of diverse diseases, such as fibrotic diseases and cancers [136–138]. Many researchers have utilized RNA sequencing, microarray analysis, and bioinformatics approaches to analyze circRNA expression profiles in various lung fibrosis models to investigate the roles of circRNAs in the regulatory networks of lung fibrosis, including IPF [139–143]. There is increasing evidence elucidating how circRNAs play regulatory roles in IPF. Table 3 shows a list of dysregulated circRNAs in IPF.

**Table 3:** The role of circRNAs in IPF.

Function	circRNA	Experimental model	Target of action	Status in IPF	Effect on IPF and pathway associated	Reference
−	circGRHPR	IPF blood and TGF-β1-induced A549 and Beas-2b cells	miR-665/NEDD4L	Down-regulated	TGFβ1-induced EMT progression of LECs	[144]
+	circ0044226	BLM fibrosis mice model and TGF-β1-induced RLE-6TN cells	CDC27	Up-regulated	EMT	[145]
		BLM fibrosis mice model	miR-7/sp1	Up-regulated	FMT and fibroblast proliferation	[156]
+	circHIPK3	SiO2-induced mouse lung fibrosis	miR-3a-3p/ FOXK2	Up-regulated	activation, proliferation, and glycolysis of fibroblasts	[148]
		BLM fibrosis mice model	miR-338-3p /SOX4, COL1A1	Up-regulated	FMT	[149]
+	circELP2	BLM fibrosis mice model	miR-1630/YAP1TAZ	Up-regulated	FMT and ECM	[151]
+	circANKRD42	IPF blood and BLM fibrosis mice model	miR-136-5p/YAP1 miR-324-5p/AJUB	Up-regulated	FMT and myofibroblast proliferation and migration	[152]
−	circSPON1	BLM fibrosis mice model	Smad3 miR-942-5p miR-52f-3p Smad7	Down-regulated	TGFβ/smad induced fibroblast activation and ECM	[157]
−	circTADA2A	BLM fibrosis mice model	miR-526b/Cav1 miR-203/Cav2	Down-regulated	activation, proliferation of fibroblast	[158]

#### circRNAs in AECs

circGRHPR has been identified as a circRNA downregulated in the peripheral blood of IPF patients and in TGF-β1-treated A549 and Beas-2b cells. Mechanistically, researchers have demonstrated that circGRHPR, by sponging miR-665, releases the E3 ubiquitin-protein ligase NEDD4-like (NEDD4L) and then promotes the ubiquitination of downstream transforming growth factor-β receptor 2 (TGFBR2), which helps reduce the responsiveness of LECs to TGF-β1 signaling. This inhibitory effect prevents the further development of TGF-β1-induced abnormal EMT and alleviating IPF [144].

Bioinformatics analysis identified that the expression of circRNA hsa_circ_0044226 was significantly upregulated in lung tissues of IPF patients. The authors propose that circ_0044226 knockdown could inhibit fibrosis primarily by the suppression of CDC27, which inhibits EMT both in vitro and in vivo and attenuates PF [145].

#### circRNAs in fibroblasts/myofibroblasts

circHIPK3 is a relatively abundant circRNA found in various human tissues [146, 147], including fibroblasts. Studies have shown that in TGF-β1-treated human lung fibroblasts in vitro, the expression of circHIPK3 is upregulated. circHIPK3 was shown to enhance the expression of FOXK2, a glycolytic transcriptional driver, by sponging miR-30a-3p and promoting fibroblast glycolysis, activation, and proliferation [148]. Furthermore, the dysregulated expression of circHIPK3 has been observed in fibroblast-to-myofibroblast transition (FMT)-derived myofibroblasts [149]. Similarly, circHIPK3 can act as an endogenous miR-338-3p sponge to regulate FMT and lead to the increased expression of SOX4 and COL1A1, which are associated with mesenchymal features and ECM.

Heterogeneous nuclear ribonucleoprotein L (hnRNP L) is a member of the hnRNP family and involves in RNA processing as an alternative RNA splicing factor [150]. Research has shown that hnRNP L initiates the backsplicing of circELP 2 and leads to its upregulated expression [151]. Researchers have demonstrated that circELP 2, through sponging miR-1630, increases Yes-associated protein 1(YAP1)/transcriptional coactivator with PDZ-binding motif (TAZ) and targets the mitochondrial quality control pathway to accelerate FMT and ECM deposition. In addition to circELP 2, Xu et al. reported that hnRNP L also participates in activating the back-splicing biosynthesis of circRNA-ankyrin repeat domain 42 (circANKRD42) [152]. circANKRD42 sponged miR-324-5p and miR-136-5p, leading to increased YAP1 entering the nucleus and YAP1 translation. Increased nuclear levels of YAP1 promote the expression of genes related to mechanical stiffness, such as Myo1c and F-actin. Recently, a study on the treatment of human umbilical cord mesenchymal stem cells (hucMSCs) further confirmed the role of the circANKRD42-YAP1 axis-mediated mechanosensing mechanism in IPF [153].

TGF-β1 is also a key factor in the regulation of PF by circRNAs. In addition to its role in AECs, circ_0044226 is upregulated in FMT-derived myofibroblasts, and miR-7 is downregulated through a sponging effect. Luciferase reporter gene analysis confirmed that sp1 (a transcription factor involved in the lung fibrosis process [154] and activation of TGF-β1 [155]) is a negative regulatory target of miR-7. Therefore circ_0044226 indirectly positively regulates the expression of sp1 and participates in the regulation of FMT and fibroblast proliferation [156]. Li et al. reported that Forkhead box protein O3 (FOXO3) can selectively promote the expression of circSPON1 and demonstrated that circSPON1 can inhibit fibroblast activation by suppressing the nuclear translocation of Smad3 induced by TGF-β1. Furthermore, circSPON1 sponges miR-520f-3p and miR-942-5p and promotes the expression of Smad7. Overall, this regulation of the TGF-β1/Smad signaling pathway ultimately inhibits the progression of PF [157]. In one study, circTADA2A was downregulated in primary human lung fibroblasts and human IPF fibroblast lines. The authors demonstrated that circTADA2A, on the one hand, upregulates the expression of caveolin-1 by sponging miR-526b, suppressing TGF-β1 signaling and fibroblast activation; on the other hand, it upregulates the expression of caveolin-2 by sponging miR-203 and thus inhibits fibroblast proliferation [158].

### The regulatory role of ncRNAs

ncRNAs play a complex and crucial role in the pathogenesis of IPF. Various types of ncRNAs not only individually drive IPF progression but also interact with each other, forming an intricate regulatory network that participates in the progression of IPF through multi-dimensional mechanisms. microRNAs represent the most extensively studied class of ncRNAs. In IPF, multiple miRNAs form an interconnected network. For instance, as previously mentioned, upregulation of miR-21 promotes pulmonary fibrosis [86], while upregulation of miR-133a attenuates fibrotic effects [90]. These two miRNAs may synergistically regulate the fibrotic process by targeting different components of the TGF-β signaling pathway. Furthermore, the ceRNA mechanism plays a pivotal role in this regulatory network. ceRNAs are RNA molecules that can mutually regulate their expression by competitively binding to miRNAs [159]. lncRNAs and circRNAs utilize this mechanism to indirectly modulate gene expression by sponging miRNAs. For example, lncRNA-ATB indirectly increases ZEB1 expression by sponging miR-200c, thereby promoting EMT [116]. Conversely, circHIPK3 can promote FMT by competitively binding miR-338-3p [149]. These ceRNA-miRNA regulatory axes occupy a significant position in the ncRNA regulatory network of IPF. The network is also closely associated with other critical signaling pathways, such as TGF-β, Wnt. For instance, the TGF-β signaling pathway can regulate the expression of multiple miRNAs, which in turn can modulate key components of the TGF-β pathway. This interaction forms a complex feedback regulatory loop in IPF. A comprehensive understanding of the complexity of this network will contribute to a more thorough comprehension of IPF pathogenesis and provide insights for the development of novel diagnostic biomarkers and therapeutic targets.

## Conclusion

**Fig. 2:**
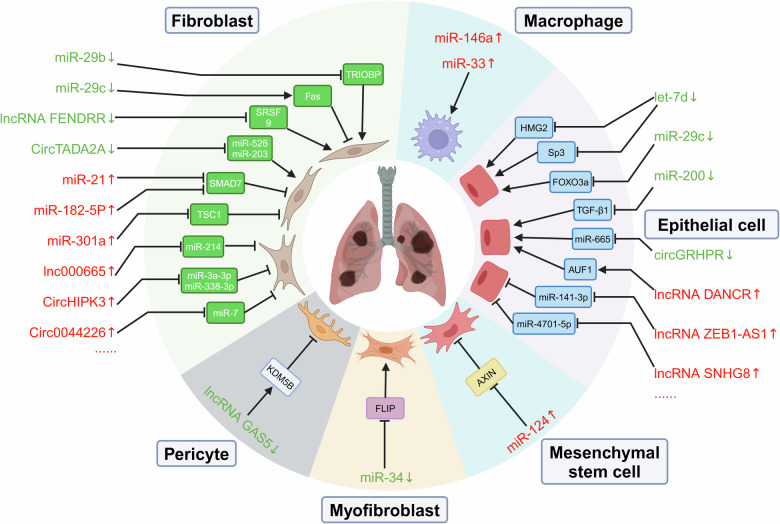
Expression changes and roles of ncRNAs in the development of IPF. The dysregulated expression of these ncRNAs can occur in various cells linked to IPF. Different types of ncRNAs target multiple downstream molecules to participate in IPF progression. (Created with BioRender.com).

ncRNAs have emerged as critical regulators of gene expression and cellular processes implicated in the pathogenesis of IPF. The dysregulation of various ncRNA species has been observed in the lungs of IPF patients and experimental models, suggesting their potential roles as drivers, mediators, or biomarkers of the disease. Expression changes and roles of ncRNAs in the development of IPF is summarized in Fig. 2. However, further research is needed to elucidate the precise mechanisms by which these ncRNAs contribute to the initiation, progression, and resolution of IPF, especially the intricate interplay between ncRNAs and their target genes, as well as the crosstalk among different ncRNA classes. The targeted modulation of dysregulated ncRNAs or their downstream effectors may offer opportunities for halting or reversing the fibrotic process, ultimately improving clinical outcomes for patients with this devastating disease.

In conclusion, the study of ncRNAs in IPF has revealed a new layer of complexity in the molecular mechanisms governing PF. Continued research efforts in this area, coupled with advancements in ncRNA delivery and targeting technologies, may pave the way for innovative approaches to combat this irreversible lung disorder.

## Data Availability

The data presented in this study are available on request from the authors.
